# Time-Varying Hazard of Patient Falls in Hospital: A Retrospective Case–Control Study

**DOI:** 10.3390/healthcare11152194

**Published:** 2023-08-03

**Authors:** Mi-Joon Lee, Bum-Jeun Seo, Myo-Youn Kim

**Affiliations:** 1 Department of Medical Information, Kongju National University, 56 Gongjudaehak-ro, Gongju-si 32588, Republic of Korea; mijoon1004@kongju.ac.kr; 2 Department of Nursing, Kangbuk Samsung Hospital, Sungkyunkwan University School of Medicine, Seoul 03181, Republic of Korea; my13.kim@samsung.com

**Keywords:** inpatients, falls, risk assessment, hospitals, survival analysis

## Abstract

This study aims to evaluate the association between patient falls and relevant factors and to quantify their effect on fall risk. This is a retrospective case–control study using the secondary data collected from a tertiary general hospital. Study subjects were 450 patients who were admitted to the hospital between January 2016 and December 2020. The prevalence of falls was associated with the fall risk level by the Morse Fall Scale (MFS) and individual status at admission including history of admission, dizziness, sleep disorder, bowel dysfunction, and urinary incontinence. The odds ratios of patient falls were higher in the low-risk group by the MFS score (odds ratio (OR) = 2.61, *p* < 0.001) and the high-risk group (OR = 5.51, *p* < 0.001) compared to the no-risk group. The hazard ratio of patient falls was higher in the high-risk group by the MFS score (hazard ratio (HR) = 3.85, *p* < 0.001). The MFS had a significant explanatory power to predict fall risk. Sleep disorder and urinary incontinence were the significant factors influencing patient falls.

## 1. Introduction

Worldwide, 684,000 people die every year due to falls, it is estimated that there are 172 million fall-related disabilities each year, and this is a global public health problem as the elderly experience the highest number of fatal falls [1].

In 2005, the World Alliance for Patient Safety published WHO draft guidelines for adverse event reporting and learning systems and there has been greater awareness of, and progress in, implementing and using patient safety reporting and learning systems in the 15 years since the draft guidelines were published [2].

The Korea Patient Safety reporting and learning system (KOPS) was introduced in 2016 to collect systematic data on patient safety incidents as the Patient Safety Act was implemented. Patient falls are also common adverse events reported in hospitals. As a result of analyzing 24,376 patient falls that were reported to the KOPS from August 2016 to December 2021, for the injury levels caused by fall, the minor injury accounted for the largest number with 13,488 cases (54.5%) followed by no injury with 7852 cases (31.7%), moderate injury with 3242 cases (13.1%), death with 122 cases (0.5%), and major injury with 42 cases (0.2%). In Korea, 1039 hospitals have assigned dedicated patient safety staff, which was 95.1% of 1092 hospitals required mandated staffing [3]. 

The United Kingdom National Health Service (NHS) calculated the total cost of inpatient falls based on the data from the National Reporting and Learning System (NRLS) between 2015 and 2016. It was estimated to be up to annually 780 million USD presenting the severity of the fall from an economic point of view [4]. 

The Joint Commission International (JCI), a globally recognized organization focused on improving healthcare quality and safety, has continually updated its National Patient Safety Goals (NPSGs) to address emerging patient safety concerns. In recent years, the JCI has shifted its attention toward patient falls as a priority area for healthcare organizations and patient falls have been identified as a distinct target in the pursuit of reducing preventable harm and enhancing patient safety [5]. As patient falls are directly related to patient safety issues, the fall incidence rate is used as a representative index to evaluate the quality of nursing care worldwide, and is a factor that affects staff burnout and patient awareness and satisfaction in hospitals [6].

Recently, fall-related litigation accounts for approximately 7% of inpatient medical litigation. Although most falls occur due to the patient’s negligence, there are often cases where nurses are held responsible. Therefore, it is important for nurses to assess fall risk factors and provide appropriate interventions [7]. Even though hospitals use various fall risk assessment tools to screen their patients at risk for falls, falls continue to occur [8]. The various fall risk assessment tools including the Morse Fall Scale (MFS), the St Thomas Risk Assessment Tool (STRATIFY), and the Hendrich II Fall Risk Model have been used in clinical settings [9,10,11] and they showed high reliability and validity in predicting the fall risk of patients [12,13,14]. 

The most commonly used fall risk assessment tool, the MFS has a high inter-measurement reliability of 0.96 and is known to be highly valid enough to predict 78% of fall patients and 83% of non-fall patients [15]. However, there have been studies showing different results regarding the use of the MFS [16]. Another study found that there were differences in the sensitivity and specificity of the MFS depending on the clinical situation [17]. Even if we use the valid assessment tool to determine the risk of falls, it is difficult to prevent all patient falls because falls are caused not only by the patient’s action but also by external factors such as hospital environments. Although fall prevention in acute hospitals is a complex and difficult problem, 78.0% of falls were predictable [18]. Therefore, it is necessary to continuously verify the validity and reliability of the MFS in various clinical environments. As seen in the previous studies, most of the fall investigations were conducted on the actual situation of falls and fall-related factors were collected by medical staff and recorded on an electronic medical record (EMR) [19]. By analyzing the stored electronic medical record data, potential risk groups can be selected and managed early to prevent falls. It can be used as basic data for intervention programs to prevent falls in the clinical field.

This study is an attempt to investigate the association between the fall-free survival time of patients and several risk factors and to evaluate the effect of time-varying factors on survival. This study is based on the inpatient falls data from electronic medical records entered by health professionals in a hospital, which is more accurate and reliable than self-reported survey data. Through a retrospective analysis of the data, we were able to identify risk factors associated with falls, and develop insights into potential improvements in existing fall prevention strategies.

## 2. Materials and Methods

This study is a retrospective case–control study using secondary data extracted from the electronic medical record and fall report system of a tertiary general hospital in Seoul. The fall report system is a software for nursing staff to report patient safety and accident to the quality management department in the hospital.

### 2.1. Fall-Related Factors and Fall Risk Assessment

The data directly related to fall event including the patient’s fall status and length of stay to fall were collected from the fall report system. Other data on factors potentially associated with patient falls acquired from the EMR system include MFS scores, clinical department, hospitalization route, length of stay, history of admission, history of surgery, dizziness, vision impairment, hearing impairment, sleep disorder, bowel dysfunction, urinary dysfunction, and BMI measured at admission.

The fall risk of individual inpatients was measured using the MFS within 24 h after admission and when the patient’s condition changed. The results of this evaluation were recorded in the EMR system. The MFS consists of the following six items in Table 1 [15] and involves the scoring of six items: fall history, presence of a secondary diagnosis, use of an ambulatory aid, use of an intravenous apparatus or heparin lock, impaired gait, and impaired mental status.

The overall score ranges from 0 to 125 points. A patient with a score less than 24 is at no risk of falling and a score between 25 and 50 means a low risk, and a score of 51 or higher indicates a high risk. 

### 2.2. Study Subjects

Study subjects were 450 patients who were admitted to a general hospital in Seoul between 1 January 2016 and 31 December 2020 and received fall risk assessment using the MFS. They were divided into a fall group who experienced at least one fall during the hospitalization period and a non-fall group without a fall. In this study, a fall was defined as an accident in which the body position was lower than the original position or to the floor due to a sudden and unintentional change in posture [20]. The case group, the fall group, included 150 randomly selected in patients who were reported to fall in the fall report system among adult patients aged 19 years or older and admitted during the study period. The control group, the non-fall group, was 300 randomly selected patients among all adult patients aged 19 years or older who were admitted to the hospital during the study period and the distribution of age and gender was matched with the case group. Pediatric patients, psychiatric patients, outpatients, intensive care unit patients, ward patients who did not receive fall risk assessment, and patients with missing records were excluded. 

### 2.3. Data Collection and Analysis

This study was conducted after obtaining the approval of the K. General Hospital Institutional review board (IRB) for the ethical protection of study subjects and informed consent forms were signed by all enrolled individuals. The purpose and method of this study were informed to subjects and it was also confirmed that all information drawn up or collected during this study would be processed with code numbers and initials so that no one could identify the personal information and all study data were stored in a computer with a strong password to allow only authorized persons to access. EMR data were obtained through the cooperation of the medical record department in the hospital. 

The data were analyzed, using SPSS Statistics version 27.0 (IBM Corp., Armonk, NY, USA). For the participants’ demographic characteristics, descriptive statistics such as frequency, percentage, mean, and standard deviation were analyzed. The differences between fall and non-fall groups based on the demographic characteristics and fall-related variables were analyzed using a chi-square test and independent two-sample *t*-test. We also used Fisher’s exact test to determine whether a statistically significant association exists between fall and relevant factors, especially in which there is any cell having an expected value 5 or less for two-way tables. Binomial logistic regression analysis was performed to evaluate the effect size of the risk factors influencing patient falls. The results were presented as odds ratio (OR) with 95% confidence intervals (CI) [21]. The discriminative power of the MFS for patient falls was estimated by the receiver-operating characteristic (ROC) area under the curve (AUC) analysis [22].

We also conducted survival analysis using the Kaplan–Meier (KM) estimator to estimate the survival function by the fraction of risk factors and to identify the time-varying covariates among factors that may contribute to falls. The KM estimator is the most common method to estimate the unadjusted probability of survival beyond a given time point. The KM curve represents the estimated survival function by plotting the survival probability between 0 and 1 against time using a step function, with each vertical descent representing the occurrence of one or more events [23]. Based on the result of KM estimation, time-dependent Cox regression was performed to obtain the hazard ratio of all risk factors with and without proportionality of hazards over time [24]. We used the method of stepwise backward removal of variables, based on a set of initial variables. The results were considered as statistically significant if it had a confidence level of 95%.

## 3. Results

### 3.1. Homogeneity for General Chracteristics between Fall Group and Non-Fall Group

Table 2 presents the result of the chi-square test for homogeneity of general characteristics between fall group and non-fall group. In terms of sex, there were 228 (50.7%) females and 222 (49.3%) males in study subjects. The average age was 68.5 (±14.9) years, of which 151 (33.6%) were 65 years and above and 299 (66.4%) under 65. There was no significant difference between fall and non-fall group for sex (*p* = 1.000) and age (*p* = 0.888) showing homogeneity. 

### 3.2. Comparison of Patient Falls Prevalence by Latent Risk Factors

Table 3 presents the characteristics of subjects including their MFS level, medical department, hospitalization route, length of stay, history of admission, history of surgery, dizziness, vision impairment, hearing impairment, sleep disorder, bowel dysfunction, urinary dysfunction, and BMI. Of the 450 participants analyzed, 11.1% had a high risk of fall in the MFS score and average length of stay was 8.85 days. Forty nine point six percent were admitted for internal medicine and 40.4% for surgical department. Sixty three point one percent were admitted through outpatient department and inpatient department, while 35.1% were admitted through emergency department. Sixty eight point four percent had at least one previous hospitalization in one year prior to this admission. Sixty one point one percent had at least one previous surgical experience in one year prior to this admission. At admission, 9.6% felt dizziness and 1.8% had impaired vision, and 7.8% had hearing impairment. Eight percent had sleep disorder and 1.6% had symptoms of bowel dysfunction, and 6.0% had urinary incontinence. Twenty four point one percent were overweight and 3.4% were obese, while 62.6% were in normal range of BMI. 

Table 3 also shows that the prevalence of fall was highest in the high-risk group (60.0%) with a high MFS score of 51 points or more followed by the low-risk group (41.5%) having a low MFS score between 25 and 50 points and the no-risk group (21.4%) with very low MFS scores of 24 points or less. The average length of stay was 11.86 days in the fall group, which is longer than 7.34 days of the non-fall group. The prevalence of falls was higher in people with experience of a previous admission to the hospital, who felt dizziness, with sleep disorder, who had bowel dysfunction, or with urinary incontinence. Clinical department, hospitalization route, history of surgery, impairment in vision and hearing, and BMI did not differ in statistical significance in the presence or absence of a fall event. 

### 3.3. Association between Physical Impairments and the Fall Risk by the MFS 

In Table 4, compared to those who had no dizziness (35.4%), the proportion of low-risk group was significantly higher in those who had dizziness (62.8%). The proportion of the high-risk group was approximately 3-fold higher in those who had sleep disorder (27.8%) than those who had not sleep disorder (9.6%). The proportion of the low-risk group was 2-fold higher in those who had urinary incontinence (74.1%) compared to those who had no urinary incontinence (35.7%). Among physical impairments, vision impairment, hearing impairment, and bowel dysfunction did not show significant association with the risk level by the MFS. 

### 3.4. Stepwise Logistic Regression to Anlalyze the Contribution of Factors to Patient Falls 

After identifying factors related to the patient’s fall risk in Table 3 and Table 4 using univariate analysis, we additionally conducted a stepwise logistic regression, which is a statistically more robust method, to examine which factors contributed most to the separation between the fall and non-fall groups. In Table 5, the final model included the MFS level, length of stay, sleep disorder, and urinary incontinence as sufficient factors affecting patient falls. The model was significant (*p* < 0.001), and had 19.7 to 27.3 percent of explanatory power for the variation in the dependent variable.

### 3.5. Odds Ratio in Logistic Regression Analysis for Patient Falls by Risk Factors 

To estimate the relationship between patient falls and the risk level by the MFS, we conducted logistic regression analysis excluding covariates unrelated to patient falls, such as medical department at admission, hospitalization route, history of admission, history of surgery, dizziness, vision impairment, hearing impairment, bowel dysfunction, and BMI of the patient. Table 6 shows that the MFS level is associated with increased patient falls risk. According to the crude model, the odds ratios of patient falls were higher in those who had a low-risk level with 25–50 points of the MFS (odds ratio (OR) = 2.61, *p* < 0.001) and a high-risk level with 51 points or more of the MFS (OR = 5.51, *p* < 0.001) compared to those who a had no-risk level with 24 points or less of the MFS. It was shown that the fall risk of the patient gradually increases (OR = 1.11, *p* < 0.001) as the length of stay increases by one day. Those who had a sleep disorder (OR = 4.57, *p* < 0.001), and urinary incontinence (OR = 7.98, *p* < 0.001) showed higher odd ratios of fall risk, which was statistically significant. After adjusting other fall-related factors such as length of stay, history of admission, dizziness, sleep disorder, and urinary incontinence, the odds ratios of patient falls were still significantly higher in those who had a low-risk level with 25–50 points on the MFS (OR = 1.95, *p* = 0.006) and a high-risk level with 51 points or more on the MFS (OR = 3.34, *p* = 0.001) compared to those who had a no-risk level with 24 points or less on the MFS. 

### 3.6. AUC Analysis for Fall Prediction of the MFS

**Figure 1:**
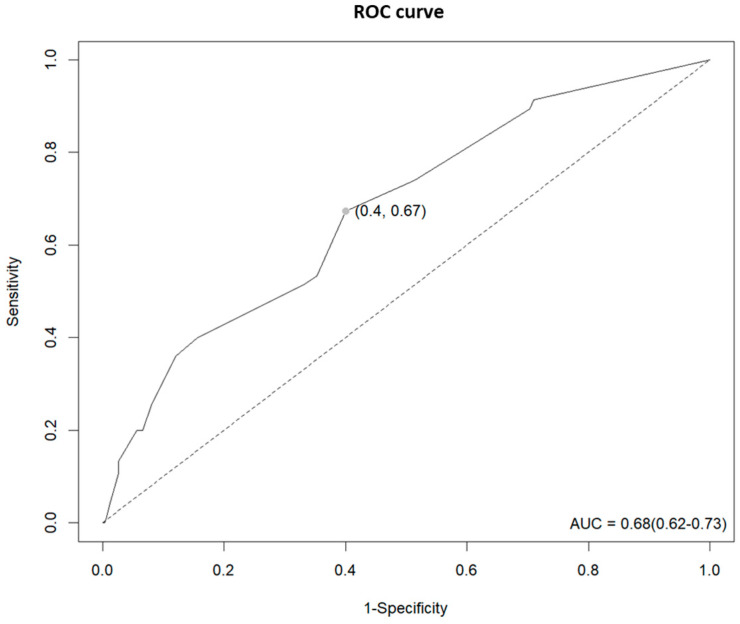
ROC curve for fall prediction of the MFS.

As can be seen in Figure 1, the MFS score was found to be an acceptable predictor of falls through the ROC curve (AUC = 0.68, 95% CI = 0.62–0.73) confirming that fall prediction using the MFS score was 68% accurate and the optimal cut-off point was 22.5. The ROC curve showed a sensitivity of 67%, a specificity of 60%, a positive predictive value positive of 45.7%, and a negative predictive value of 78.6%.

### 3.7. Kaplan–Meier Estimates for the Fall-Free Survival Probability against Time by Risk Factors

**Figure 2:**
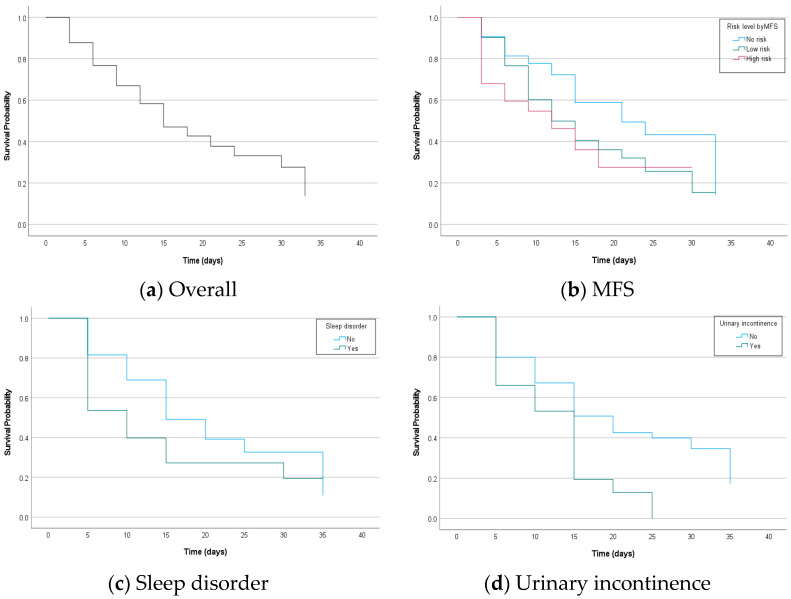
Kaplan–Meier curve displaying the estimated survival probability for patient falls against time (in days) according to (**a**) overall, (**b**) risk level by the MFS, (**c**) whether or not to have sleep disorder, and (**d**) whether or not to have urinary incontinence.

Figure 2 shows the KM curves of the estimated survival probability for patient falls against time by risk factors. The median fall-free survival time for all patients was 14 days (interquartile range (IQR) 6–31 days). Regarding the risk level by MFS scores, patients in the no-risk group had a significantly better median fall-free survival (23 days; IQR 11–31 days) compared to patients in the low-risk group (12 days; IQR 6–29 days) and in the high-risk group (10 days; IQR 2 days-upper range not calculable secondary to censored data). The difference in fall-free survival probability against time between risk groups by the MFS was statistically significant (Log-rank chi-square 15.540; *p* < 0.001). It was presented that patients who had a sleep disorder at admission had a statistically significant worse median fall-free survival (7 days; IQR 1–29 days) compared to patients did not feel dizziness (15 days; IQR 8–31 days) and the difference in fall-free survival probability was statistically significant (Log-rank chi-square 9.847; *p* = 0.002). Patients who had urinary incontinence at admission had a statistically significant worse median fall-free survival (10 days; IQR 3–13 days) compared to patients who did not feel dizziness (16 days; IQR 7–31 days) and the difference in fall-free survival probability was statistically significant (Log-rank chi-square 12.777; *p* < 0.001).

### 3.8. Hazard Ratio in Time-Dependent Cox Regression Analysis for Patient Falls by Risk Factors

In survival studies to measure the effect of time-varying covariates for the outcome event such as death, diagnosis, or fall, the Cox proportional hazard model has been used widely. However, the associations between the survival outcome and time dependent covariates may be biased unless they meet the proportional hazard (PH) assumption. The PH assumption in the Cox model means that the hazards should be proportional, which means that the relative hazard remains constant over time for different covariate levels and the most common way to measure the proportional hazard assumption is visual assessment of KM curves [25]. It was assessed whether the KM survival curves by the level of factors crossed in at least one of the presented Figure 2 and all factors including the risk level by the MFS, sleep disorder, and urinary incontinence did not satisfy the PH assumption as their KM curves intersected within the study period. Therefore, we performed time-dependent Cox regression to reflect the time-varying effect of factors by adjusting time in Cox regression model [26]. 

The factors remaining in Table 7 showed significant associations with patient falls as risk predictors. In unadjusted model, the hazard ratio of patient falls was higher in those who were in the high-risk group by MFS scores (hazard ratio (HR) = 3.85, *p* < 0.001) compared to those who were in the no-risk group. Those who were in the low-risk group also showed a higher hazard ratio of patient falls (HR = 1.48, *p* = 0.154) but it was not statistically significant. Length of stay did not show significant association. The hazard ratio of patient falls was higher in those who had a sleep disorder (HR = 3.84, *p* < 0.001) and urinary incontinence (HR = 2.24, *p* = 0.001). After adjusting the effect from other factors, the hazard ratio of patient falls is still significantly higher in those who were in the high-risk group by MFS scores (HR = 4.56, *p* < 0.001) compared to those who were in the no-risk group. Those who were in the low-risk group also showed a higher hazard ratio of patient falls (HR = 1.52, *p* = 0.135) but it was still not statistically significant. As the length of stay increased, the fall risk slightly decreased and it was significant (HR = 0.94, *p* < 0.001). The hazard ratio for patient falls was significantly higher in those who had a sleep disorder (HR = 2.23, *p* = 0.001) and urinary incontinence (HR = 2.22, *p* = 0.001). 

## 4. Discussion

The epidemiological design, retrospective case–control of this study has great validity and precision to determine the association between patient falls and risk factors as exposures, since the exposures were evaluated at the moment of admission, not having to resort to the memory of an individual. Furthermore, our findings revealed that initial fall risk assessments during the admission process are important as they were shown that the risk levels by fall assessment have a significant association with the prevalence of falls in high-risk patients. Our study also provided important insights into the time-varying effect of fall-related factors to highlight the importance of continuous prevention plans across the hospitalization period. We also explored the relevant factors affecting the occurrence of patient falls in hospitals and these findings are expected to provide useful information to prevent fall at the point of care. We expect that these results can be used to estimate how likely individual patients will fall in the hospital.

When we conducted homogeneity test for the general characteristics of participants, there were no significant differences in sex and age between the fall and non-fall groups. This result illustrates that it is possible to estimate the association between patient falls and risk factors excluding the potential biases caused by the confounding effects of age and gender [27]. In this study, approximately 33% of the participants experienced a fall and it is similar to the results of previous studies, which found that falls continue to be the number one adverse event with approximately 2–30% of inpatients falling at least once during their hospitalization [28] and approximately 30% of falls in people aged 65 years or older in the US annually [29,30]. 

As a result of univariate analysis using a chi-square test comparing the prevalence of fall event by the level of potential fall-related factors, it was shown that the prevalence of patient falls was significantly associated with the risk factors including the risk level by MFS scores, length of stay, history of admission, dizziness, sleep disorder, and urinary incontinence. In the present study, the higher the MFS scores when the patient is admitted, the higher the incidence rate of falls during their hospitalization, which was consistent with the findings of previous studies [31,32]. These findings illustrate that the MFS is a comprehensive fall risk assessment tool to predict the probability of inpatient falls in hospitals causing not only healthcare costs increase but also severe interference of a patient’s quality of life. Therefore, we suggest that hospitals keep implementing inpatient falls prevention and monitoring programs based on valid and reliable methods. 

This study reported that the fall group had a longer length of stay with 11.86 days than the non-fall group with 7.34 days, which was comparable to the result of the previous study that the faller group tended to be with a longer hospital stay [33]. On the other hand, there was a previous study showing a converse relationship that the extended length of hospital stay was due to patient falls [34]. Therefore, we estimated the relative odds and relative hazard in patient falls according to the length of stay in hospital. The odds ratios of patient falls were significantly higher in those who stayed longer but the hazard ratios were not significantly higher, which was similar to the result of a previous study [35]. 

Dizziness is a general term that can be described as several sensations, including spinning or non-spinning vertigo, disequilibrium, lightheadedness, floating, or a combination thereof. It is necessary to inform patients and caregivers about dizziness and the possibility of falls caused by dizziness at admission. In this study, there was a significant difference in dizziness between the fall group and the non-fall group. For patients who have felt dizziness, it is necessary to re-evaluate their fall risk more frequently than patients with other risk factors and pay attention to the underlying treatment as there is a previous study showing that dizziness is associated with functional disability and risk of falls [36]. 

This study showed that the prevalence of falls was associated with a patient’s health status such as sleep disorder. It is similar to the results of previous studies that as many as 50% of older adults reported sleep problems and sleep disturbances were associated with an increased risk of falls excluding the effect of confounders [37,38]. These findings suggest that programs to maintain adequate sleep quality during admission should be developed based on the individual’s sleep problems.

In this study, it was shown that patients with urinary incontinence are more likely to fall than patients without it, which is consistent with the results of previous studies [39,40]. Based on the findings, when allocating a patient’s bed, it is recommended to place it near the toilet or provide a portable toilet to minimize the patient’s movement, and fall prevention education should be provided to care for patients with urinary incontinence. 

Using the Kaplan–Meier survival estimates, we found that there was a significant difference in survival of patient falls by the risk level from MFS scores, sleep disorder, and urinary incontinence, which was consistent with the results of previous studies [41,42,43]. Furthermore, the time-dependent Cox regression analysis referring previous studies at which time-dependent explanatory variables are measured [44,45] indicated that the four covariates including the risk level by the MFS, length of stay, sleep disorder, and urinary incontinence were still significantly associated with patient falls events after explicitly adjusting for the times.

In this study, the optimal cut-off point on the MFS that best distinguishes between people with and without fall risk was 22.5 and it is close to the current classification criteria of the MFS score of 25 points to divide the no-risk and the low-risk groups. However, there were previous studies that have investigated the most optimal MFS score for predicting fall events using AUC analysis and resulted their optimal scores were ranged from 45 to 55 points [46,47,48]. Hence, the current MFS score of 25 points, which is the criterion for the low-risk group, may not be appropriate for classifying the fall risk and these findings are consistent with the result of this study that only high-risk group showed a significant difference in hazard ratio from the no-risk group.

This study has potential limitations. First, since the effect estimates in this study were based on the data obtained from a single tertiary hospital, external validity may be insufficient to generalize the results to all hospitals. Second, although the control group (those without the outcome) was selected to have the same characteristics as the case group, there may be bias due to not considering the homogeneity in health status or other socioeconomic factors except age and gender. Third, this study did not take into account the impact of individual differences in the capabilities of medical staff, such as nurses who investigate fall risk and risk-related factors at admission. Fourth, there was a paucity of previous studies to be cited regarding patient falls risk using time-dependent Cox regression analysis, which may hinder the ability to contextualize and validate out findings. Despite these limitations, it is expected that further studies on the time-varying effect of fall risk factors would be actively conducted based on our study results.

## 5. Conclusions

In conclusion, through this study, it was confirmed that the MFS had a significant explanatory power for fall risk of inpatients and other factors such as sleep disorder and urinary incontinence could be used as indicators to measure fall risk more precisely along with the MFS. By improving fall prevention strategies, healthcare facilities can reduce healthcare costs, enhance patient safety, and improve the overall quality of care provided to their patients. Furthermore, more effective education and nursing intervention programs for fall prevention can be developed using these findings.

## Figures and Tables

**Table 1:** The Morse Fall Scale.

Item	Scale	
1. History of falling: immediate or within 3 months	No	0
	Yes	25
2. Secondary diagnosis	No	0
	Yes	15
3. Ambulatory aid		
Bed rest/nurse assist		0
Crutches/cane/walker		15
Furniture		30
4. IV ^1^/Heparin lock	No	0
	Yes	20
5. Gait/Transferring		
Normal/bedrest/immobile		0
Weak		10
Impaired		20
6. Mental status		
Oriented to own ability		0
Forget limitations		15

^1^ IV: Intravenous therapy.

**Table 2:** Homogeneity test for general characteristics between fall group and non-fall group.

Variable	Total	Fall (*n* = 150)	Non-Fall (*n* = 300)	χ^2^, t	*p*-Value
	*n* (%), *Mean ± SD*	*n* (%), *Mean ± SD*	*n* (%), *Mean ± SD*		
Sex				0.000	1.000
Male	222 (49.3)	74 (33.3)	148 (66.7)		
Female	228 (50.7)	76 (33.3)	152 (66.7)		
Age ^1^	68.5 ± 14.9	68.9 ± 15.0	68.3 ± 14.8	0.403	0.687
<65	151 (33.6)	51 (33.8)	100 (66.2)	0.020	0.888
≥65	299 (66.4)	99 (33.1)	200 (66.9)		

^1^ Units expressed as years.

**Table 3:** Comparison of patient falls prevalence by latent risk factors.

Variable	Total	Fall (*n* = 150)	Non-Fall (*n* = 300)	χ^2^, t	*p*-Value
	*n* (%), *Mean ± SD*	*n* (%), *Mean ± SD*	*n* (%), *Mean ± SD*		
MFS ^1^	25.82 ± 20.87	34.53 ± 21.49	21.47 ± 10.15	−6.548 ***	<0.001
No risk (0–24)	229 (50.9)	49 (21.4)	180 (78.6)	35.839 ***	<0.001
Low risk (25–50)	171 (38.0)	71 (41.5)	100 (58.5)		
High risk (≥51)	50 (11.1)	30 (60.0)	20 (40.0)		
Length of stay ^2^	8.85 ± 6.71	11.86 ± 7.04	7.34 ± 6.011	−7.089 ***	<0.001
Department				5.209	0.074
Internal	223 (49.6)	63 (28.3)	160 (71.7)		
Surgical	182 (40.4)	69 (37.9)	113 (62.1)		
Others	45 (10.0)	18 (40.0)	27 (60.0)		
Hospitalization route ^3^				1.923	0.382
ED	158 (35.1)	56 (35.4)	102 (64.6)		
OPD/IPD	284 (63.1)	93 (32.7)	191 (67.3)		
Others	8 (1.8)	1 (12.5)	7 (87.5)		
History of admission				9.512 **	0.002
No	142 (31.6)	33 (23.2)	109 (76.8)		
Yes	308 (68.4)	117 (38.0)	191 (62.0)		
History of surgery				2.263	0.133
No	175 (38.9)	51 (29.1)	124 (70.9)		
Yes	275 (61.1)	99 (36.0)	176 (64.0)		
Dizziness				6.801 **	0.009
No	407 (90.4)	128 (31.4)	279 (68.6)		
Yes	43 (9.6)	22 (51.2)	21 (48.8)		
Vision impairment					0.450
No	442 (98.2)	146 (33.0)	296 (67.0)		
Yes	8 (1.8)	4 (50.0)	4 (50.0)		
Hearing impairment				1.549	0.211
No	415 (92.2)	135 (32.5)	280 (67.5)		
Yes	35 (7.8)	15 (42.9)	20 (57.1)		
Sleep disorder				19.565 ***	<0.001
No	414 (92.0)	126 (30.4)	288 (69.6)		
Yes	36 (8.0)	24 (66.7)	12 (33.3)		
Bowel dysfunction					<0.001
No	443 (98.4)	143 (32.3)	300 (67.7)		
Yes	7 (1.6)	7 (100.0)	0 (0.0)		
Urinary incontinence				25.532 ***	<0.001
No	423 (94.0)	129 (30.5)	294 (69.5)		
Yes	27 (6.0)	21 (77.8)	6 (22.2)		
BMI ^4^	22.96 ± 3.69	23.29 ± 3.84	22.79 ± 3.61	−1.363	0.174
Underweight	44 (9.9)	15 (34.1)	29 (65.9)	4.115	0.249
Normal	278 (62.6)	85 (30.6)	193 (69.4)		
Overweight	107 (24.1)	44 (41.1)	63 (58.9)		
Obesity	15 (3.4)	6 (40.0)	9 (60.0)		

** *p* < 0.01, *** *p* < 0.001. ^1^ Units expressed as scores and no risk (0–24), low risk (25–50), high risk (≥51). ^2^ Units expressed as days. ^3^ ED: emergency department, OPD: outpatient department, IPD: inpatient department. ^4^ Units expressed as kg/m^2^ and underweight (<18.5), normal (18.5–24.9), overweight (25.0–29.9), Obesity (≥30.0).

**Table 4:** Association between physical impairments and the fall risk by the MFS.

Variable	No Risk (*n* = 229)	Low Risk (*n* = 171)	High Risk (*n* = 50)	χ^2^, t	*p*-Value
	*n* (%)	*n* (%)	*n* (%)		
Dizziness				13.684 **	0.001
No	218 (53.5)	144 (35.4)	45 (11.1)		
Yes	11 (25.6)	27 (62.8)	5 (11.6)		
Vision impairment				4.912	0.086
No	227 (51.4)	165 (37.3)	50 (11.3)		
Yes	2 (25.0)	6 (75.0)	0 (0.0)		
Hearing impairment				4.201	0.122
No	217 (52.3)	153 (36.9)	45 (10.8)		
Yes	12 (34.3)	18 (51.4)	5 (14.3)		
Sleep disorder				11.499 **	0.003
No	216 (52.2)	158 (38.2)	40 (9.6)		
Yes	13 (36.1)	13 (36.1)	10 (27.8)		
Bowel dysfunction				0.285	0.869
No	225 (50.8)	169 (38.1)	49 (11.1)		
Yes	4 (57.1)	2 (28.6)	1 (14.3)		
Urinary incontinence				19.122 ***	<0.001
No	226 (53.4)	151 (35.7)	46 (10.9)		
Yes	3 (11.1)	20 (74.1)	4 (14.8)		

** *p* < 0.01, *** *p* < 0.001.

**Table 5:** Stepwise logistic regression analysis for the contribution of factors to patient falls.

Variable	*B*	*S.E.*	Wald	df	*p*-Value	exp (*B*)
MFS ^1^						
No risk (0–24)						
Low risk (25–50)	0.679	0.244	7.765	1.000	0.005 **	1.97
High risk (≥51)	1.196	0.355	11.355	1.000	0.001 **	3.31
Length of stay ^2^	0.080	0.017	22.356	1.000	<0.001 ***	1.08
Sleep disorder						
No						
Yes	1.291	0.400	10.437	1.000	0.001 **	3.64
Urinary incontinence						
No						
Yes	1.704	0.502	11.518	1.000	0.001 **	5.49
Goodness of fit						
Cox&Snell R^2^/Nagelkerke R^2^				0.197/0.273		
χ^2^(df), *p*-value				97.577(7), <0.001		

** *p* < 0.01, *** *p* < 0.001. ^1^ Units expressed as scores and no risk (0–24), low risk (25–50), and high risk (≥51). ^2^ Units expressed as days.

**Table 6:** Logistic regression analysis for patient falls according to the risk level by the MFS (*n* = 450).

Variable	Unadjusted OR ^1^		Adjusted OR ^2^	
	OR (95% CI)	*p*-Value	OR (95% CI)	*p*-Value
MFS ^3^				
No risk (0–24)				
Low risk (25–50)	2.61 (1.68–4.04) ***	<0.001	1.95 (1.21–3.13) **	0.006
High risk (≥ 51)	5.51 (2.88–10.53) ***	<0.001	3.34 (1.66–6.70) **	0.001
Length of stay ^4^	1.11 (1.07–1.14) ***	<0.001	1.08 (1.05–1.12) ***	<0.001
Sleep disorder				
No				
Yes	4.57 (2.22–9.43) ***	<0.001	3.70 (1.69–8.10) **	0.001
Urinary incontinence				
No				
Yes	7.98 (3.15–20.23) ***	<0.001	5.61 (2.10–15.02) **	0.001

** *p* < 0.01, *** *p* < 0.001. OR: odds ratio for hypertension. CI: confidence interval. ^1^ Unadjusted OR: includes only the risk level by the MFS. ^2^ Adjusted OR: adjusted for covariates including length of stay, sleep disorder, and urinary incontinence. ^3^ Units expressed as scores and no risk (0–24), low risk (25–50), high risk (≥51). ^4^ Units expressed as days.

**Table 7:** Time-dependent Cox regression analysis for patient falls (*n* = 450).

Variable	Unadjusted HR ^1^		Adjusted HR ^2^	
	HR (95% CI)	*p*-Value	HR (95% CI)	*p*-Value
MFS ^3^				
No risk (0–24)				
Low risk (25–50)	1.48 (0.86–2.53)	0.154	1.52 (0.88–2.63)	0.135
High risk (≥51)	3.85 (1.99–7.46) ***	<0.001	4.56 (2.31–9.00) ***	<0.001
Length of stay ^4^	1.00 (0.96–1.04)	0.927	0.94 (0.91–0.97) ***	<0.001
Sleep disorder				
No				
Yes	3.84 (2.11–6.98) ***	<0.001	2.23 (1.41–3.54) **	0.001
Urinary incontinence				
No				
Yes	2.24 (1.41–3.56) **	0.001	2.22 (1.36–3.63) **	0.001

** *p* < 0.01, *** *p* < 0.001. HR: hazard ratio for patient falls. CI: confidence interval. ^1^ Unadjusted HR: includes only time and the risk level by the MFS. ^2^ Adjusted HR: adjusted for time and covariates including length of stay, sleep disorder, and urinary incontinence. ^3^ Units expressed as scores and no risk (0–24), low risk (25–50), high risk (≥51). ^4^ Units expressed as days.

## Data Availability

The data presented in this study are available on request from the corresponding author.
